# Exploring Stakeholder Roles and Strategies for Preventing Teenage Pregnancy: A Comprehensive Analysis in Lepelle‐Nkumpi District: South Africa

**DOI:** 10.1111/hex.70219

**Published:** 2025-03-15

**Authors:** Rakgadi Grace Malapela, Sibusiso Memory Zuma, Patrone Rebecca Risenga

**Affiliations:** ^1^ Department of Health Studies College of Human Sciences, University of South Africa Pretoria South Africa

**Keywords:** comprehensive analysis, exploring, non‐governmental organisations, preventing, roles, strategies, teenage pregnancy

## Abstract

**Introduction:**

Trends and trace of teenage pregnancy continue to rise, and this poses a threat to the future of the education of the youth, as well as their well‐being—ultimately the economic status of the country. The aim of this study is to gain insights from non‐governmental organisations about the strategies to curb teenage pregnancy in Lepelle‐Nkumpi District of Limpopo province.

**Methods:**

An exploratory qualitative research design was utilised to obtain insiders' perspectives about the strategies to curb teenage pregnancy. Thirty non‐governmental organisation workers were purposively selected from the five sub‐districts in Capricorn District, Limpopo province. Five focus groups, conducted in English, each lasted one to 2 h. A thematic approach was used—analysis method was used to deductively code, interpret and summarise data into themes.

**Results:**

Five themes emerged such as multi‐stakeholder collaborations, economic empowerment, information seeking and sharing sessions, nurturing bonds with children, and extra mural activities.

**Conclusion:**

The study highlighted the need for multifaceted approaches and collaborations to prevent teenage pregnancy. This helps policymakers, healthcare providers, educators, stakeholders, and researchers create strategies to reduce teenage pregnancy rates. This supports the United Nations Sustainable Development Goals: good health and well‐being, quality education and gender equality.

**Public Contributions:**

The study participants were members of non‐governmental organisations, who shared their lived experiences. The information gathered was analysed and interpreted to reach conclusions, without involving the participants in the data analysis and interpretation process.

## 1 Introduction

Teenage pregnancy is a significant global health concern [1, 2] and a multilayered issue [3]. It is associated with a high burden of sexual, reproductive, health and rights problems including coerced sex, teenage pregnancies, early marriage [4] and sexually transmitted infections (STIs) [5]. In the review conducted by [2], it was noted that while around two‐thirds of young individuals abstain from sexual activity before the age of 16, by the time they reach 20, approximately 85% of them will have engaged in vaginal intercourse. This underscores the need for comprehensive relationships and sexuality education (RSE) and accessible services to prevent unintended pregnancies and promote sexual health amongst young people.

On the other hand, the World Health Organization (WHO) emphasised that developing regions, including South Africa, face significant challenges concerning teenage pregnancy [6]. Approximately 12 million girls aged between 15 and 19, and at least 777,000 girls under the age of 15, give birth annually in these regions. Additionally, complications arising from pregnancy and childbirth are major contributors to mortality amongst girls between those ages worldwide. Teenage pregnancy has detrimental effects on the health, education, and economic well‐being of young people, and it perpetuates these challenges over time [1, 7]. Moreover, it is evident that teenagers with limited education or from poor backgrounds are at a higher risk of experiencing teenage pregnancy [1]. Therefore, it is imperative to prioritise research in rural villages, where the population is confronted with higher levels of disadvantage and encounters additional barriers.

The estimated global adolescent birth rate for girls aged between 10 and 14 years in 2022 was 1.5 per 1000 women. However, higher rates were observed in Latin America, the Caribbean (2.4), and sub‐Saharan Africa, including South Africa (4.6) [6]. Numerous challenges hinder adolescents from accessing and utilising contraceptives effectively to prevent unintended pregnancies [7]. As of 2022, the global proportion of women aged between 15 and 49, who have their family planning needs met with modern contraceptive methods (SDG indicator 3.7.1) stood at 77.5%, indicating a 10‐percentage point increase since 1990 (67%) [8].

The slow progress in increasing contraceptive use can be attributed to various factors, including limited options for contraceptive methods, and restricted access to services, especially among young, poor, and unmarried individuals. Additionally, fear or experience of side‐effects, cultural or religious opposition, inadequate quality of available services, biases amongst users and providers regarding certain methods and gender‐based barriers contribute to the challenges [7, 8].

According to a study by [9], early marriage, love/relationship dynamics, premarital sex, sexual assault (rape) and low educational status emerged as the most significant determinants of teenage pregnancy. Traditional practices of early marriage prevalent in communities, religious restrictions on contraception, and lack of economic support were identified as factors making teenage girls particularly vulnerable to pregnancy [10].

The WHO collaborates with stakeholders to advocate for addressing teenage pregnancy, building an evidence base, developing supportive policies and programme tools, facilitating capacity building and providing country support [7]. However, in Panama, strategies intended to promote healthier behaviour and capacity development amongst young people, including HIV and STI prevention and intimate partner violence prevention, have demonstrated limited effectiveness due to issues such as inadequate implementation, low sustainability, and insufficient knowledge or skills amongst implementers [11]. This research took place in a rural village in the Lepelle‐Nkumpi District of Limpopo province, South Africa. This study involved the collaboration of non‐governmental organisations (NGOs) to gather important information about the roles and strategies needed to tackle teenage pregnancy in that setting. The engagement of these NGOs contributed important perspectives to tackle the issue of teenage pregnancy in the rural village. Despite the presence of interventions, strategies, and policies advocating for the prevention of teenage pregnancy, the challenge of high teenage pregnancy rates persists [4, 6, 7, 8, 12, 13]. Consequently, this prompted the researchers to undertake this endeavour in response to the appeal made by a NGO, highlighting the prevalent issue of numerous learners facing teenage pregnancy. The data concerning teenage pregnancy extends beyond mere figures, encapsulating a harsh reality experienced by certain girls attending schools in South Africa [14]. Research carried out in India has revealed that teenage pregnancy carries lifelong and intergenerational health consequences, significantly impacting a significant portion of the adolescent female population. This underscores the need for a comprehensive exploration of the factors that contribute to adolescent pregnancy and its outcomes [1]. A previous study conducted in the Ekurhuleni District of South Africa focused on teenagers aged between 13 and 19 years. The study emphasised the importance of prevention strategies that go beyond addressing risky sexual behaviour. It recommended a deeper understanding of teenagers' experiences and the circumstances leading to pregnancy, which would provide valuable insights to enhance pregnancy prevention programmes [14]. Previous research conducted in Northern Uganda and South Africa has highlighted the urgent need for interventions to address and reduce the prevalence of teenage pregnancy, with the aim of preventing further increases [15, 16]. This is particularly crucial considering that teenage pregnancies are predominantly unintended. Considering this, the present study focused on collaborating with various NGOs in the Lepelle‐Nkumpi district to gather their perspectives on effective strategies for preventing teenage pregnancy. It is worth noting that there is a scarcity of research conducted in the rural villages of Limpopo province that actively engages NGOs in addressing this issue.

The primary focus of this study was to gather information on the strategies employed to prevent teenage pregnancy in the rural villages of Lepelle‐Nkumpi district, located within the Limpopo province. The main research question posed to participants was to provide an overview of these prevention strategies. Subsequently, probing questions were used to delve deeper into the roles of various stakeholders in implementing these strategies and to identify potential strategies that could be developed to address teenage pregnancy.

To guide the investigation, the study adopted the Social Cognitive Theory framework, which allowed for an exploration of the behavioural, personal, and environmental factors that influence the effectiveness of preventive strategies against teenage pregnancy. The aim was to gain valuable insights from NGOs regarding their experiences and expertise in curbing teenage pregnancy within the Lepelle‐Nkumpi district of the Limpopo province.

### 1.1 Theory

The research study utilised the Social Cognitive Theory as its framework, aiming to comprehensively comprehend the interplay between behavioural, personal and environmental factors in relation to the roles and strategies employed in preventing teenage pregnancy. By adopting this framework, the researchers sought to explore the influence of various social, economic, and political factors on prevention efforts. The works of [17] as well as [18] provided valuable insights and informed the decision to utilise the Social Cognitive Theory in this study. Social Cognitive Theory plays a crucial role in preventing teenage pregnancy by highlighting the influential factors within various social contexts. This theory emphasises the importance of schools, NGOs, churches, communities, and community health workers in shaping attitudes and behaviours. By leveraging these social structures, the theory helps identify and address the key elements that contribute to effective strategies for the prevention of teenage pregnancy.

### 1.2 Purpose

The purpose of this study was to gain insights from NGOs about the strategies to curb teenage pregnancy in Lepelle‐Nkumpi district of Limpopo province.

### 1.3 Objectives

→ To explore the roles of stakeholders in the prevention of teenage pregnancy in rural villages of Lepelle‐Nkumpi district, Limpopo province.
→ To recommend strategies to prevent teenage pregnancy in rural villages of Lepelle‐Nkumpi district, Limpopo province.

## 2 Methods and Design

### 2.1 Sample

A total of 30 members from NGOs actively involved in providing both community and school‐based Sexual and Reproductive Health Services (SRHSs) were selected as participants for this study. The participants were divided into five groups, with each group consisting of five members. These five NGOs were specifically chosen from the Lepelle‐Nkumpi District.

In accordance with the recommendations made by [19], it was deemed appropriate and feasible to have group discussions with a range of 3–15 members. The recruitment of participants took place over a 3‐month period, from March to June 2023.

### 2.2 Setting

**Figure 1:**
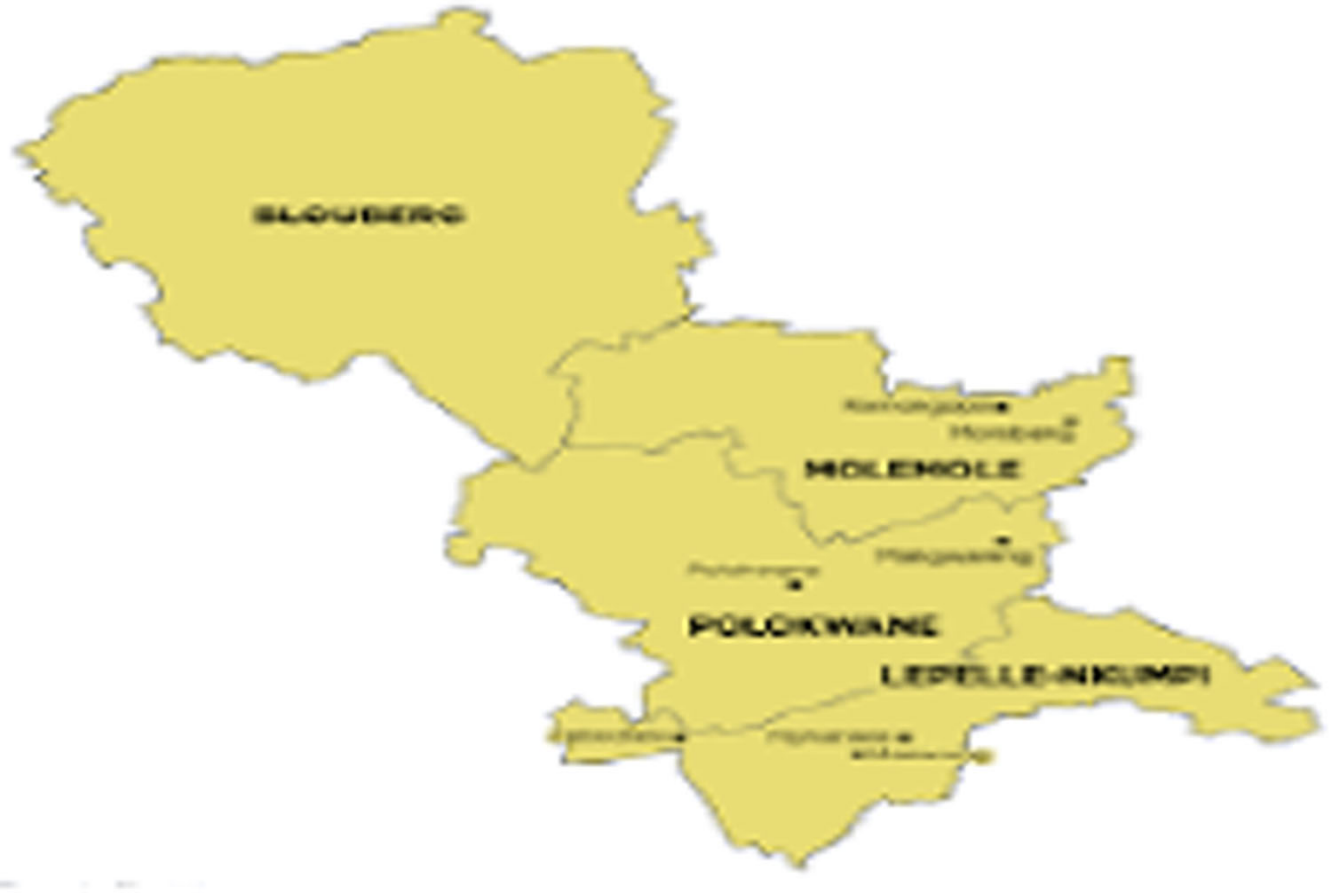
Capricorn district map [21].

The research was conducted in the Lepelle‐Nkumpi sub‐district, which is part of the Capricorn district in the Limpopo province. The Capricorn district comprises of four sub‐districts: Blouberg, Molemole, Lepelle‐Nkumpi and Polokwane [20]. The specific choice to focus on Lepelle Nkumpi was influenced by a community‐driven call from NGOs that highlighted the presence of pregnant learners across all school grades in the area. Additionally, this decision aligned with the objectives outlined in the [12], particularly Strategic Thrust 1, which aims to promote the overall well‐being of young people. The policy specifically targets youth who are at risk of pregnancy, sexual assault, abuse, substance abuse, and emotional disturbances. A visual representation of the Capricorn District and the Lepelle‐Nkumpi sub‐district is depicted in Figure 1 below.

### 2.3 Research Design

A qualitative exploratory descriptive study was conducted to gather insights from NGOs about various strategies that can be employed to prevent teenage pregnancy. Face‐to‐face focus group discussions were conducted, with each group consisting of four to five members. A discussion guide was utilised to ensure consistency and facilitate the discussions. The guide was initially presented in English and clarified in the local vernacular language (Sepedi) whenever necessary, as the participants had a good command of English but required clarification in certain instances. Sepedi was chosen as it was the most spoken language in the district, and the researchers were proficient in it, helping to address any language barriers.

Before the study, a pretest of the data collection instrument was conducted with one group of five members, but the results of this pretest were not included in the final analysis. Each focus group was given 1 h to discuss and document their thoughts on strategies to prevent teenage pregnancy. Participants were also encouraged to express their views orally and in writing. To ensure comprehensive data collection, the discussions were audio recorded to avoid missing any information. Data collection took place over 3 days, from 20 to 23 June 2023. Data saturation was achieved after the fourth focus group, but the researchers decided to include one more group to ensure the collection of in‐depth and exhaustive information until no new insights emerged.

### 2.4 Sampling Criteria

The study employed purposive and snowball sampling methods, which are non‐probability sampling techniques, to select the NGOs that participated in the research. Additionally, referrals were used to recruit additional NGOs. This approach allowed the study to gather insights from a diverse range of NGOs actively engaged in youth empowerment programmes, including initiatives focused on preventing teenage pregnancy within the community. A total of 30 members from these NGOs participated in the study.

### 2.5 Ethical Consideration

The study obtained an ethical certificate from the University College of Human Research Ethics, valid from 29 April 2021 to 29 April 2024, with the reference number Rec‐240816‐052. Before seeking permission from the NGOs involved, the study received approval from the appropriate committee. Participation was voluntary with verbal and written consent obtained. Participants could withdraw anytime. Research was conducted in a private conference room to ensure confidentiality. Focus groups were numbered to maintain anonymity.

### 2.6 Data Analysis

The Braun and Clarke six steps of data analysis was used to transcribe data, code and corroborate the findings into themes and sub‐themes [19, 22]. These steps included: (1) Transcribing the data; (2) data familiarisation; (3) coding; (4) theme development; (5) data interpretation: defining themes and categories; and (6) data presentation. To add value to the data analysis, the Qualitative Assisted Data Analysis Software (QADAS) was used following ATLAS TI version 2023, themes and sub‐themes emerged.

## 3 Research Findings

The study involved a total of 30 participants. Many participants were females within the age range of 30–39 years and had completed their education up to Grade 12. Regarding work experience, most participants had accumulated 5–6 years of professional experience. The socio‐demographic profile of the participants is presented in Table 1 below, outlining these key characteristics.

**Table 1:** Socio‐demographic profile of the participants (30).

Characteristics	Frequency	Percentage
Age range		
20–29 years	12	40%
30–39 years	17	57%
40–49 years	1	3%
Gender		
Female	26	87%
Male	4	13%
Highest qualification		
Grade 12	19	63%%
Certificate	5	17%
Diploma	3	10%
Degree	3	10%
Working of working experience with the community		
1–2 years	0	0%
2–3 years	5	17%
3–4 years	6	20%
5–6 years	14	46%
7–8 years	5	17%
Capricorn district		
Polokwane sub‐district	1	3%
Lepelle‐Nkumpi sub‐district	29	97%
Non‐governmental Organisations (NGOs)		
NGO 1	10	33%
NGO 2	4	13%
NGO 3	2	7
NGO 4	6	20%
NGO 5	8	27%

### 3.1 Themes and Sub‐Themes

Six themes emerged, which included multi‐stakeholder collaborations, access to health services, information seeking and sharing sessions, economic empowerment, nurturing bonds with children and extramural activities. Refer to Table 2 below for the summarised themes and sub‐themes.

**Table 2:** Summarised themes and sub‐themes.

Themes	Sub‐themes
Theme 1: Multi‐stakeholder collaborations	1.1 Integrating religious values with comprehensive guidance 1.2 Collaborative efforts for teenage pregnancy prevention 1.3 Sexual education and support system 1.4 Healthy family relationships and open dialogues 1.5 Health awareness and accessibility
Theme 2: Information seeking and sharing sessions	2.1 *Mitigating the risks of teenage pregnancy*
Theme 3: Economic empowerment	3.1 Prevention of poverty
Theme 4: Nurturing bonds with children	4.1 Positive role modelling
Theme 5: Extramural activities	5.1 Involvement in sports and recreation

### 3.2 Multiple‐Stakeholder Collaborations

Participants mentioned the need for collaborations with various stakeholders to address teenage pregnancy which is a major challenge in the community. The following was quoted:

“There should be more campaigns at schools and churches where there are youth. NGOs must do research in support of other stakeholders and try to collaborate with them. Moshate (Kingdom) should collaborate with NGOs and stakeholders to host the Imbizo (gathering). Councillors should engage in this teenage pregnancy by bringing love and life programmes to the communities. Churches must also have a youth day where they address teenage pregnancy to educate the youth about sex ‐ they should not preach or teach the word from the Bible only.”

FGDs 2

"NGOs to form partnerships between parents, churches and community to help teenagers and have their best interests at heart.”

FGDs 4

The five sub‐themes were as follows:

#### 3.2.1 Balancing Religion and Comprehensive Guidance

Participants mentioned that the church also need to play their part and not preach on the concentrating of spiritual and religious aspects only. This was shared with the following quotes:

“Churches: Biblical aspects, sex before marriage is a sin, and they should promote kids from abstaining from sex. They preach abstinence until marriage and do teaching for young and girls and boy son how to maintain virginity and wait for the right time according to the bible”

FGDs 1

“Churches: Must not focus too much on the word, they must preach about abstaining from sex and make youth programmes. To preach sex education, educate for policies and programmes that can address the root of teenage pregnancy. Create a safe space for open discussions about sex, promote abstinence. Offer support and guidance to teenagers facing reproductive health challenges”

FGDs 2

#### 3.2.2 Collaborative Efforts to Teenage Pregnancy Prevention

The quotes to support the collaborative efforts from the communities were as follows:

“Schools, healthcare institutions, community health workers, families, community and churches must promote and have information accessible to run awareness and put in place all tools acquired to inform children about teenage pregnancy. They do campaigns, engage with schools and make sure every community has clinics and social workers and counsellors. NGOs do event whereby they teach about sex and distribute condoms”.

FGDs1

“Communities and others must support the NGOs with donations so that they can help the poor to reduce teen pregnancy and poverty because they are doing it in exchange for food. They do awareness campaigns about sex education”

FGDs 2

“Raises awareness on gender‐based violence, prevention of HIV/AIDS, sexually transmitted illnesses when teens are raped. School visits to motivate and educate learners about sex, teenage pregnancy, substance abuse and family to build a relationship between learners and teachers”

FGDs 3

#### 3.2.3 Support System and Sexual Education

Participants highlighted that the schools need to provide sexual education and act as a support system. These are the sub‐themes and quotations that emerged from the findings.

Support system

“We need support agents at schools (social workers). School to invite health facilities to do health awareness campaigns from different institutions community‐based organizations (CBOs) and Non‐Governmental Organizations (NGOs).”

FGDs 2

“They have Early Childhood Development (ECD) organization that goes into schools.”

FGDs 3

Sexual education

“Life orientation programmes and schools must be used as a tool to educate learners about teenage pregnancy. Educate the learners about the risk of teenage pregnancy. Partnering with stakeholders to bring awareness, e.g., Department of Health, Social Development and Non‐Governmental Organizations (NGOs).”

FGDs 1

“Educate teenagers about teenage pregnancy, including its disadvantages and advantages. They have subjects such as life orientation that educates children about reproduction, etc.”

FGDs 3

"All schools should enforce programs that involve support, tutoring, education, and recreation, as these programs can help reduce high risk. Strengthen health, sex education and extramural activities. They invite social workers”

FGDs 4

“Education by having sex talks and not making it a taboo subject, laying out the consequences of teenage pregnancy, and learning to respect yourself.”

FGDs 5

#### 3.2.4 Healthy Family Relations and Open Dialogues

To encourage healthy family relations and open dialogues. It was uttered by the following quotes:

Healthy family relations

“Parent/guardian should encourage/teach their children about sex. Family problems should not affect children as parents. we must give our children love and more time. To provide support and guidance on sexual education. To take their children for contraceptives”

FGD 2

“Parent involvement is considered necessary in teen pregnancy prevention and preventing other adolescent risky behaviours. Parents should also educate and examine their children regarding teenage pregnancy.”

FGDs 4

“Be supportive and learn to listen to teenagers, be open, and communicate. Act as support agents to teenagers and give them hope.”

FGDs 3

Open dialogues

“Parents and guardians should normalise talking about sexual health education with their children.”

FGDs 1

“Having honest and open conversations about sex. Listening to teenagers”

FGDs 4

“Families should encourage teenagers. Be clear about your own sexual values and attitudes. Talk with your children early and often about sex and love. You should know your children's friends and their families. Discourage early, frequent, and steady dating.”

FGDs 5

#### 3.2.5 Health Awareness and Widen Access to Sexual and Reproductive Health Services

The participants mentioned that healthcare institutions and community health workers should raise health awareness. Access to health services should be maximised, and it would be beneficial to prevent teenage pregnancies. The supporting quotations were as follows:

“Give access and be free to access health services. Home‐based carers must be accessible and practice confidentiality. NGOs and community health workers be trained to facilitate prevention methods. Motivational talks on the prevention of teenage pregnancy. Provide mobile clinics in school with NGOs partnering with health researchers.”

FGDs 3

“Teen needs regular healthcare services to receive comprehensive sexual and reproductive health counselling. Nurses can educate and advise young people about sexually transmitted diseases and contraception. Promote sex education programmes. Educate about sexuality. Increase the use of contraceptives”

FGDs 4

“They must go to the schools to provide contraceptives and provide information.”

FGDs 5

### 3.3 Information Seeking and Sharing Sessions

The study participants alluded to several strategies to address the issue of forced sex, early marriages, media influence, and discouragement. Participants also mentioned that forced sex is a grave violation of human rights and can contribute to early marriages, which have numerous negative consequences, including increased risk of teenage pregnancy.

The quotes to support the information‐seeking and sharing sessions were as follows:

“We have programs like Soul City on TV that teach teenagers about health and pregnancy and how to live your life. Using slogans like ‘No to teenage pregnancy, teenage pregnancy must fall”

FGDs 1

“Community dialogues and girl talks on sexual reproductive health rights.”

FGDs 2

“Computer laboratories and libraries to seek information. Continue to raise awareness in communities and schools. Also, allow those who want to have access to healthcare services for prevention to receive services while at school.”

FGDs 3

“Take the help of a fictional character or literature to get your teenager interested in the conversation.”

FGDs 5

#### 3.3.1 Mitigating the Risks of Teenage Pregnancy

Participants identified several factors linked to teenage pregnancy that require attention. These were highlighted by the following quotes:

“Reduce forced sex. Prevent early marriages and monitor the influence of the media. Provide career counselling and abstinence education. Forced sex is a grave violation of human rights and can contribute to early marriages, which have numerous negative consequences, including an increased risk of a teenage pregnancy. Social media has a huge influence on teenage pregnancy because they see pregnancy content. It gives them access to pornography and they want to try it out.”

FGDs 4

“Time on social networks should be limited and monitored to avoid too much exposure to pornography and sexual content.”

FGDs 2

### 3.4 Economic Empowerment

Participants indicated that government economic empowerment initiatives can prevent teenage pregnancy, often stemming from vulnerable backgrounds. Financial relationships with older men increase the risk of unintended pregnancies, highlighting the need for such initiatives to support at‐risk teenagers.

“Economic empowerment, the government needs to empower young people economically to prevent teenage pregnancy from occuring because of their family history. They find themselves in relationships with old men for money. Teaching our girls to do things for themselves and do entrepreneurship in schools, so our girls grow knowing that even if our government/country has a lack of jobs. They will have knowledge about opening their businesses and be taught to do things for themselves, not depend on men. There is too much exposure to sexual content. Social media has influences who post a luxury lifestyle, and most teenagers believe that they must sleep with older men to access the lifestyle.”

FGDs 1

“Absorb teenagers in the workspaces during the holidays to equip them. A budget allocation from the government specifically for teenage programs.”

FGDs 4

“Address the socio‐economic issues through community engagement.”

FGDs 2

#### 3.4.1 Prevention of Poverty

The participants indicated that teenage pregnancy contributes to ongoing poverty and increased school dropout rates, thereby restricting teenagers' prospects for a brighter future and better career opportunities

“The phenomenon of teenage pregnancy has devastating social and economic costs. Early pregnancy in South Africa forces many girls to drop out of school and trap others in a cycle of poverty, leaving most stigmatized by society for being a teenage mother or forced into early marriages. Therefore, addressing socioeconomic issues through community involvement is important.”

FGDs 2

“Establishing economic empowerment and different programmes for adolescents to keep them engaged.”

FGDs 4

### 3.5 Nurturing Bonds With Children

Participants stressed that promoting gender equality and strong relationships with children fosters healthy environments. Encouraging teenagers to challenge stereotypes, providing diverse role models, and maintaining open, non‐judgmental communication are crucial. Positive role models inspire children to adopt these values

“Encourage conversations and activities that show that interests, skills, and goals are not gender‐specific to inspire children to confront and question prevailing gender stereotypes.”

FGDs 3

“Promote gender equality and develop a good relationship with children. It is important to create open channels of communication with children to allow them to express thoughts, feelings, and concerns they are experiencing.”

FGDs 4

“Select an appropriate time to talk preferably when your teen is free from other distractions.”

FGDs 5

#### 3.5.1 Positive Role Modelling

By being a positive role model, you can inspire children to emulate these values in their own lives.

“Give them examples of people who defy gender stereotypes and a variety of role models from the media and literature, as well as people in their community. Parental trainings for parents to play their role, stand up for themselves and enforce discipline.”

FGDs 3

### 3.6 Extramural Activities

#### 3.6.1 Involvement in Sports and Recreation

Participants described extramural activities and said it refers to organised activities that take place outside of the regular school curriculum. These activities can include sports, clubs, community service, art programmes, and other recreational or educational pursuits. Extramural activities provide teenagers with opportunities to engage in positive and constructive pursuits. By participating in sports, clubs, or other activities, teenagers can divert their focus and energy toward activities that promote personal growth, skill development and social interaction. This can help reduce the likelihood of engaging in risky behaviours, such as early sexual activity and unprotected sex, which can lead to teenage pregnancy. Below, is the quotation that supports the use of extramural activities as an alternative to prevent teenage pregnancy:

“Sport facilities and recreational centres where teenagers can have access to extramural activities should be used. More recreational activities to prevent children from engaging in sexual activities. These activities must also be monitored and ensure that children are participating and have no sex.”

FGDs 3

“Sports, clubs, or other activities can help teenagers to divert their focus and energy towards activities that promote personal growth, skill development, and social interaction.”

FGDs 1

“Establish safe spaces within the community for more sports facilities.”

FGDs 4

## 4 Discussion

The aim of this study was to gather insights from NGOs regarding effective strategies for reducing teenage pregnancy in the Lepelle‐Nkumpi District of Limpopo province. According to the NGOs, they strongly advocate for collaborative efforts involving multiple stakeholders to effectively address this issue. They emphasise the importance of strategies such as improving access to health services, organising information‐seeking and sharing sessions, mitigating risks of adolescent pregnancy, promoting economic empowerment, fostering nurturing relationships with children and encouraging participation in extramural activities. These strategies are considered essential in preventing teenage pregnancy within rural villages. In line with the Social Cognitive Theory, these strategies address behavioural factors by providing individuals with the necessary resources, skills, and opportunities to make informed decisions about their sexual and reproductive health. They also consider personal factors by empowering individuals through economic opportunities and fostering nurturing relationships, which can positively influence their self‐efficacy and self‐regulation in making healthy choices. Furthermore, the emphasis on multi‐stakeholder collaborations and mitigating the risks of teenage pregnancy acknowledges the role of environmental factors, including social, economic, and political influences, in shaping the context within which teenage pregnancy prevention efforts take place.

### 4.1 Multi‐Stakeholder Collaborations

Multi‐stakeholder collaborations have gained prominence in addressing the health needs of adolescents, as guided by the Global Accelerated Action for the Health of Adolescents (AA‐HA!) framework by the [6]. These collaborations assist rural villages, districts, provinces, NGOs, governments, and education and health systems in determining their plans and approaches to adolescent health. Teachers and Community‐Based Health Workers (CBHWs) have been identified as key actors in addressing adolescent sexual and reproductive health (ASRHR) issues. Their roles include community mobilisation, providing SRHR counselling to adolescents and guardians, and facilitating referrals to SRHR services when necessary [4]. However, challenges such as stigmatisation associated with sexual abuse and pregnancy, shyness amongst girls during SRHR discussions with boys present, and misconceptions about contraception have been reported. A systematic literature review conducted in Low‐ and Middle‐Income Countries (LMICs), including South Africa, supports the importance of stakeholder collaboration and involvement in the design and appropriateness of Comprehensive Sexual Education (CSE) interventions [5]. The review highlights the need for key players in teaching to be involved for successful implementation of CSE, along with building strong relationships between teachers and community actors, considering political, social, legal, and financial factors. Supplementing the regular educational curriculum with empowerment programmes, as suggested by [23] in Panama, may lead to healthier relationships and reduced rates of teenage pregnancies. The study conducted in Uganda emphasises the importance of collective responsibility and concern within the community to prevent teenage pregnancy [18]. The findings of a study conducted in the Northwest province of South Africa emphasised the importance of involving external stakeholders in supporting teachers and learners to prevent teenage pregnancy, highlighting the need for their active engagement beyond the classroom [24]. This responsibility includes parents, family members, teachers, health personnel, political and religious leaders and other community service providers who need to be equipped with sexual and reproductive health knowledge to enhance the fight against teen pregnancies. According to the study conducted in Philippines, mothers and older family members arose as salient facilitators by assisting adolescents navigate access to initial Long‐Acting Reversible Contraception (LARC) use [25].

A scoping review conducted by [26] highlights the need for concerted efforts in service re‐organisation to ensure access to sexual and reproductive health services during the COVID‐19 pandemic. These services include antenatal care, postnatal care, contraception, safe abortion care, and clinical management of rape survivors. In India, a study found that adolescent girls often lack awareness of family planning methods, which can be attributed to illiteracy [9]. Even if they are aware, they may not utilise family‐planning services due to limited accessibility. Improving access to effective contraception is crucial in preventing teenage pregnancies, aligning with the principles of the You're Welcome standards [2].

The findings of these studies address specific targets [27] related to promoting health and well‐being [7]. The goal is to ensure universal access to sexual and reproductive healthcare services, including family planning, education, and integration of reproductive health into national strategies and programmes by 2030. A study in Uganda revealed the importance of community‐based health facilities that provide a friendly, private, and safe environment for sexually active teenagers to receive timely services and free contraceptives to prevent unwanted pregnancies [18].

In urban areas of South Africa, participants emphasised the need for visible campaigns addressing teenage pregnancy [28]. These campaigns could involve billboards, TV and radio advertising, and school‐based initiatives to promote youth‐friendly services at the primary healthcare level. Accessible and youth‐friendly contraceptive services are crucial in encouraging early uptake of advice [2]. A study in Tanzania found that the failure to obtain free contraceptives and the lack of privacy when seeking them may discourage girls from using contraceptives, leading them to rely on unreliable methods [29].

Support from healthcare personnel, including guidance on contraception, is essential, and health campaigns should target both teachers and secondary school learners [30]. In South Africa, the idea of establishing a school‐based contraceptive clinic (SBCC) received support from parents and focus group participants, but resource and logistical constraints pose challenges to its implementation [31]. Recommendations by [32] emphasise the need to address the accessibility of youth‐friendly family planning and reproductive health services.

### 4.2 Information Seeking and Sharing Sessions

Several studies conducted in different countries have provided valuable insights in support of promoting the information seeking and sharing session for addressing challenges related to adolescent sexual and reproductive health.

In Zambia, it was suggested that creating safe spaces for adolescents to discuss sexual and reproductive health issues, as well as involving them in finding solutions are essential. This approach empowers adolescents to actively participate in the decision‐making processes regarding their own health [4]. However, the study also highlighted the importance of considering cultural norms when utilising mass media for sexual education, as these norms may hinder open discussions between parents and children [33].

In Uganda, accurate information and education on contraceptives were emphasised as crucial for sexually active adolescents to make informed choices. Additionally, the study recommended the support of government and other stakeholders in implementing adolescent‐friendly interventions, such as accessible information, counselling, and comprehensive sexual and reproductive health services [18]. Similarly, in Mexico, reinforcing educational initiatives on sexual and reproductive health for adolescents was deemed necessary [34].

Recognising the importance of gender equality, another study emphasised the need to direct reproductive health education efforts towards empowering boys in the same manner as girls [35]. In Uganda, it was suggested that law enforcement against sexual abuse amongst girls could significantly contribute to improving adolescent sexual and health services in low‐income setting [36].

### 4.3 Nurturing Bonds With Children

Participants in the study believe that a strong bond between parents and children plays a crucial role in preventing teenage pregnancy. Hadley [2] supports this view, emphasising the importance of an open and honest culture around sex and relationships, which is associated with lower teenage pregnancy rates. Partnerships with parents and carers should be fostered to address knowledge gaps about fertility, concerns about contraceptive side effects, and support young people in choosing and using their preferred method. Positive modelling behaviour by family and peers is also protective against teenage pregnancy amongst adolescent males [37].

### 4.4 Economic Empowerment

Participants in the studies also highlighted the importance of economic empowerment in preventing teenage pregnancy. A study conducted in Pakistan found that region and socioeconomic status, including education, occupation, and wealth index, were key determinants of teenage pregnancy [38]. In India, it was recommended to address women's rights and the needs of marginalised and vulnerable women, along with efforts focused on improving early marriage, education, and empowering women and girls [1]. Generation skills training and provision of resources, such as seeds or animals for rearing, have been successful strategies implemented by NGOs in rural communities, helping them generate income and overcome financial crises, thereby supporting the financial needs of teenagers [18]. Another study conducted in Nigeria concluded that economic empowerment is crucial as empowered girls are better prepared to handle reproductive health issues. It was also suggested that religious bodies, parents and schools should provide counselling and guidance to promote positive reproductive and sexual health behaviours amongst teenagers [39]. The Nigerian study further emphasised the need for government action to combat poverty at all levels as a strategy to curb teenage pregnancy [40]. Strengthening interventions aimed at increasing economic independence amongst teenagers, reducing child marriage, and promoting contraceptive utilisation amongst married teenagers were highlighted as effective measures to address the issue of teenage pregnancy [41].

### 4.5 Extramural Activities

Participants in the study believe that engaging in extramural activities, such as recreation and sports, can serve as an alternative method to prevent teenage pregnancy. A study conducted in Uganda highlighted the need for innovative programmes to minimise the risk of pregnancy from sex trade amongst in‐school teenage girls during the COVID‐19 pandemic [42]. Therefore, recreation and sports can provide a productive and healthy outlet for teenagers, potentially reducing their engagement in risky sexual behaviours.

## 5 Implications for Research, Schools, Communities, Clinical Practice and Policymakers

Strong collaborations between schools, healthcare systems, communities, NGOs, and families are crucial to prevent teenage pregnancy. Schools must provide comprehensive sexual education, while families must engage in open discussions to break taboos. Health services should be tailored to meet the needs of teenagers, increasing access to youth‐friendly care. Communities and NGOs can promote positive role models and entrepreneurial skills to promote self‐reliance and reduce poverty. More recreational facilities are needed to engage teens, reducing sexual risky behaviours. Policymakers should support further research on improving youth‐friendly services, ensuring that policies are informed by evidence to better address adolescents' health and social needs.

## 6 Strengths and Limitations

The study was only limited to one district in Limpopo province. The strength of this study lies in collecting data from various NGOs. However, findings of the study cannot be used generalised due its contextual limitations.

## 7 Conclusion

The aim of this study was to gather insights from NGOs on strategies to prevent teenage pregnancy in the Lepelle‐Nkumpi district of Limpopo province. The research highlighted the importance of collaborative efforts involving multiple stakeholders, as it was recognised that a single solution cannot adequately address the diverse needs of both male and female youth. Specifically, five key stakeholders were identified as crucial contributors to the prevention of teenage pregnancy: schools, NGOs, churches, communities and community health workers.

The study highlights the importance of increasing access to sexual and reproductive health services and suggests that schools should play a key role in providing comprehensive sexual education. Strong collaborations between the health system, parents, and NGOs were identified as essential for preventing teenage pregnancy. Empowering disadvantaged families and promoting extramural activities were also seen as crucial preventive measures, reducing risky sexual behaviours. The findings support the Social Cognitive Theory, emphasising the interaction between behavioural, personal, and environmental factors in addressing teenage pregnancy. These insights offer valuable guidance for policymakers, communities, and stakeholders in rural areas like the Lepelle‐Nkumpi District.

## Author Contributions

**Rakgadi Grace Malapela:** conceptualization, data curation, formal analysis, visualization, writing – original draft, methodology, writing – review and editing, software, resources, project administration. **Sibusiso Memory Zuma:** conceptualization, methodology, data curation, formal analysis, writing – review and editing, writing – original draft. **Patrone Rebecca Risenga:** conceptualization, data curation, formal analysis, writing – original draft, writing – review and editing, supervision.

## Ethics Statement

The study adhered to ethical guidelines, obtaining approval from the research ethics committee. Participants were adults who voluntarily consented, with the freedom to withdraw. Confidentiality was maintained using numbers. Researchers employed bracketing to mitigate bias and preconceptions during data collection.

## Conflicts of Interest

The authors declare no conflicts of interest.

## Data Availability

The data that supports the findings of this study are available from the corresponding author upon reasonable request.
